# Sport-Specific Testing and Training Methods in Youth

**DOI:** 10.3390/sports14030103

**Published:** 2026-03-04

**Authors:** Alessandra Amato, Andrea Fusco, Cristina Cortis

**Affiliations:** 1 Department of Biomedical and Biotechnological Sciences, Section of Anatomy, Histology and Movement Science, School of Medicine, University of Catania, Via S. Sofia n°97, 95123 Catania, Italy; alessandra.amato@unict.it; 2 Department of Medicine and Aging Sciences, University “G. d’Annunzio” of Chieti-Pescara, 66100 Chieti, Italy; 3 Department of Human Sciences, Society and Health, University of Cassino and Lazio Meridionale, 03043 Cassino, Italy; c.cortis@unicas.it; 4 European University of Technology EUt+, 03043 Cassino, Italy

## 1. Introduction

Research on the best athletic performance is undergoing a paradigm shift, characterized by a progressive decline in the age of specialization in numerous sports [1,2,3,4], while providing the opportunity for accelerated skill acquisition. This trend also presents a significant challenge for sports scientists, coaches, and physicians. The common belief among coaches, parents, and young athletes is that early and year-round specialization in a single sport can lead to elite performance and may also have a different influence on the development of a specific skill that affects everyday life, such as manual dexterity [5,6]. However, a growing number of studies contradict this theory, suggesting that such a limited approach may instead restrict children’s exposure to diverse sporting experiences, impair the development of fundamental motor skills, and compromise the foundations for an active lifestyle [7,8,9]. In addition, recent studies have suggested that early specialization is associated with an increased risk of injury in various sports, for both boys and girls, as well as burnout and dropping out [10,11]. The key principle underlying this Special Issue is that young athletes represent a distinct population, not simply miniature adults. Their biological and psychological landscape is defined by ongoing processes of growth, maturation, and development, which profoundly influence their responses to training, susceptibility to injury, and long-term athletic outcomes [12,13,14]. The evidence consistently shows that the plastic neuromuscular system of a child or adolescent responds differently to aerobic and anaerobic stimuli than that of mature athletes. There are periods of accelerated adaptation, often conceptualized as “windows of opportunity” or sensitive periods, for components such as speed, strength, and skill acquisition [14,15]. Furthermore, the immature skeletal system, with the presence of growth cartilage, is more vulnerable to overload injuries, particularly under the repetitive high-load volumes typical of early specialization [16,17]. From a psychological perspective, the increasingly professionalized youth sports environment can contribute to burnout and anxiety, necessitating approaches that balance performance development with psychological well-being [18,19]. For these reasons, increasingly original research is investigating training and assessment strategies designed and adapted specifically for young athletes in different sports disciplines. For example, in a sport such as volleyball, recent studies have shown that differential training can improve technique and performance in the spike-jump, even in young athletes, highlighting the importance of interventions targeted at the specific technical and biomechanical needs of athletes with specific characteristics [20]. This Special Issue has brought together contemporary approaches to the management of young athletes, with a focus on the adaptation and specificity of methodologies, prioritizing the physiological and psychological demands of this population. Thirteen original articles have been published, generally classified into two key areas: training, which focuses on the application of specific interventions to improve athletic performance in young athletes, and testing, which explores the tools and methods used to assess physical abilities, monitor load, and validate new protocols in youth populations.

## 2. An Overview of the Published Articles

Results from training interventions demonstrate the impact of structured sport-specific training on young athletes, highlighting that specific programs can cause significant improvements in both physical and cognitive domains. Teteris et al. (Contribution 1) provide a neurophysiological perspective, showing that a 12-week reaction training program can enhance the choice reaction time in under-20 fencers. This is particularly relevant in sports where split-second decisions are a key skill and underscores the trainability of cognitive functions alongside physical skills during a crucial developmental period. On the other hand, Vasilescu and colleagues (Contribution 2) provide a reproducible evidence-based intervention tailored to the neuromotor development of children aged 10 to 12 years, designed specifically to improve lower limb strength in junior combat sports, recognizing that explosive strength is crucial for performing technical and decisive actions during combat. The intervention also addresses the essential need for training to be tailored to be effective. In basketball, three studies offer complementary insights. Amato et al. (Contribution 3) compared core and mobility training in youth players, finding that both 8-week interventions effectively improved the dynamic balance, overhead squat depth, and agility, pointing to the specific neuromuscular benefits of such training. However, Sansone et al. (Contribution 4) centered their research on another pivotal factor influencing basketball performance: the pre-competition warm-up. The study entailed a detailed comparative analysis of two distinct warm-up protocols administered to the same cohort of adolescent basketball athletes, demonstrating that warm-ups incorporating small-sided games more accurately replicate sport-specific match conditions. However, the findings also underscore the necessity of monitoring the external load parameters and ensuring sufficient physiological readiness within such protocols. This investigation thus yields practical evidence-based guidance for optimizing warm-up strategies in 16-year-old basketball players. On a longer timeline, Castro-Collado et al. (Contribution 5) demonstrated the holistic benefits of a structured school-based basketball program over 38 weeks. Their findings revealed not only improved physical fitness (a 45% increase in aerobic capacity) but also positive changes in body composition in prepubertal boys, advocating for such programs as a public health and athletic development tool. The importance of specificity is further emphasized by Herbert et al. (Contribution 6) in another team sport: handball. Their 11-week professional training program for elite adolescent players led to significant gains in sport-specific defense and offense times, with under 17 players also showing remarkable improvements in the VO_2_ peak, ball velocity, and jump height. This study powerfully argues that targeted sport-specific training during adolescence is a key point for a professional career. Sagat similarly demonstrated how structuring specific sports training, such as sprint intervals with change of direction in soccer players, could significantly improve the explosive power, agility, speed, anaerobic capacity, and maximal oxygen uptake (VO_2_max) in male under-17 soccer players (Contribution 7). Finally, Perazzetti et al. (Contribution 8) shifted the focus to the critical aspect of recovery and load management. Monitoring under-16 water polo players during a congested match schedule, they found that the internal load was correlated with increased fatigue and decreased well-being, highlighting how recovery is a crucial part of training.

These papers focused on training offer a balanced approach between the importance of planning adequate training in terms of volume and also scheduling adequate recovery, which is crucial in this phase of growth for developing technical and physical skills. Reliable and valid testing is the foundation through which effective training is built. Other contributions of our Special Issue focused on advancements in testing and assessment, validated new protocols, compared testing environments, and explored the underlying physiological and biomechanical factors of performance. A key practical question was addressed by Carron et al. (Contribution 9), who found that run-based performance tests (sprints, agility, and fitness) in adolescent rugby league players yielded comparable results on both indoor and outdoor surfaces, offering valuable flexibility for practitioners working in varied environments. Despot and Plavec (Contribution 10) made a significant contribution by validating a new Ballroom Aerobic Test (BAT) for dance sport couples. The BAT showed strong agreement with gold-standard laboratory tests, providing a feasible and accurate field-based and sport-specific method to assess the aerobic capacity of dancers, a population often overlooked in sport science. The theme of specificity continues in the work of Crisafulli et al. (Contribution 11), who described the power–load relationship in elite canoeists and kayakers. Their data on bench press, ballistic bench press, and prone bench pull exercises provide coaches with preliminary benchmarks for designing strength and power training tailored to the unique “pull-dominant” demands of paddling sports. At a more fundamental physiological level, Massini et al. (Contribution 12) investigated the agreement between different high-intensity endurance indexes (Critical Speed, Respiratory Compensation Point, and 50%Δ) in young male runners. Their finding that these indices are broadly similar, though not entirely interchangeable at an individual level, refines our understanding of how to demarcate and target key training intensities in youth running. Two studies examine the long-term development of athletes. Marcelli et al. (Contribution 13) explored the relative age effect in a youth soccer academy, confirming that the birth month can influence motor performance and ball skills within annual age groups. This highlights the need for individualized training programs that account for biological maturity and not just chronological age. Meanwhile, Ouergui et al. (Contribution 14) demonstrated that interlimb asymmetry and bilateral deficit indexes measured in vertical jumps are significant predictors of sport-specific kicking performance in taekwondo athletes. This underscores the importance of balanced strength development and targeted neuromuscular training, even in sports dominated by unilateral actions.

The papers aimed to explore testing in this population, highlighting the importance of assessing by means of field-based and sport-specific methods before planning specific sports training and training load progression and the need to make the assessment more practical and applicable even outside laboratory settings, which may differ from real-world performance.

## 3. Conclusions

The findings of this Special Issue underline that the effective management of the young athlete requires a specific and dual-focused approach. It demands training that is not only structured and intense but also cognitively engaging, sport-specific, and carefully balanced with recovery. It equally depends on testing that is valid, reliable, practical, and sensitive to the specific demands of the sport and the individual characteristics of the developing athlete. The career path of a young athlete is a complex mix of growth, training, and competition. The research presented here provides coaches, athletic trainers, and sports scientists with insights and tools that are essential for approaching this path more effectively. Given the success of this Special Issue, we have launched a second edition and hope to receive contributions focusing on other sports so that we can provide an increasingly comprehensive and specific overview of the metabolic and technical requirements of different disciplines, whether they are individual or team sports, indoor or outdoor, power or endurance performance. This will allow us to deepen our knowledge in these areas and to better achieve our goal: to promote an environment that improves performance, ensures well-being, and cultivates a lifelong passion for sport.
